# Online Soft Sensor of Humidity in PEM Fuel Cell Based on Dynamic Partial Least Squares

**DOI:** 10.1155/2013/923901

**Published:** 2013-12-17

**Authors:** Rong Long, Qihong Chen, Liyan Zhang, Longhua Ma, Shuhai Quan

**Affiliations:** ^1^School of Automation, Wuhan University of Technology, Wuhan, Hubei 430070, China; ^2^College of Science, Huazhong Agricultural University, Wuhan, Hubei 430070, China; ^3^School of Information Science and Engineering, Ningbo Institute of Technology, Zhejiang University, Ningbo, Zhejiang 315100, China

## Abstract

Online monitoring humidity in the proton exchange membrane (PEM) fuel cell is an important issue in maintaining proper membrane humidity. The cost and size of existing sensors for monitoring humidity are prohibitive for online measurements. Online prediction of humidity using readily available measured data would be beneficial to water management. In this paper, a novel soft sensor method based on dynamic partial least squares (DPLS) regression is proposed and applied to humidity prediction in PEM fuel cell. In order to obtain data of humidity and test the feasibility of the proposed DPLS-based soft sensor a hardware-in-the-loop (HIL) test system is constructed. The time lag of the DPLS-based soft sensor is selected as 30 by comparing the root-mean-square error in different time lag. The performance of the proposed DPLS-based soft sensor is demonstrated by experimental results.

## 1. Introduction

Proton exchange membrane (PEM) fuel cells utilize the chemical energy from the reaction of hydrogen and oxygen to produce electricity, water, and heat. They have advantages such as faster start-up, high power density, low emissions, high efficiency, a simple design, safe operation, and environmental friendliness. As alternative power generators, PEM fuel cells are the most suitable for transport applications and portable power generation [1]. Maintaining proper membrane humidity is one of the key requirements for PEM fuel cell to reach its optimum performance because ionic conductivity depends critically on the hydration levels. Specifically, greater hydration will result in greater conductivity and thus a more efficient cell. However, excess hydration levels will result in a layer of liquid water to be formed and a variety of performance and durability problems [2], including voltage loss at high current density due to porous passages to be blocked by liquid water, voltage instability, unreliable start-up under freezing conditions, and so forth [3].

Online monitoring humidity in the fuel cell is an important problem in maintaining proper membrane humidity. But the cost and size of existing humidity sensors are prohibitive for online measurements. In situ visualization is essential for a better understanding of liquid water in PEM fuel cell. Several techniques have been developed for visualization of liquid water inside the membrane electrode assembly (MEA) [4, 5]. These visualization techniques mainly include direct visualization [6, 7], magnetic resonance imaging (MRI) [8], neutron radiography [9, 10], and X-ray imaging techniques. Among these techniques, the direct visualization has the advantage of providing high temporal and spatial resolution information about water transport in the gas flow channels. But the PEM fuel cell system should have a transparent window to facilitate optical observation. MRI is a widely available, inherently three-dimensional output data and capable of visualizing water in opaque structures. MRI is employed to the in-plane direction of a PEM fuel cell and observed the formation and slow propagation of a dehydration front from the gas inlet side to the gas outlet side of the cell. Neutron radiography is highly sensitive to water and a well-established technique for studying the water distribution in the MEA. In-plane neutron imaging of an operating PEM fuel cell is employed and produced a time series of images to evaluate the water management of a fuel cell system. The X-ray image technique can give the temporal and spatial resolutions, especially that the use of synchrotron radiation makes it capable to reach higher spatial resolutions.

In situ visualization technique can detect the liquid water in PEM fuel cell and convert into membrane humidity. However, the equipment used in these techniques is usually valuable and is not economic to measure the membrane humidity online when PEM fuel cell is acted as power sources of portable applications. Soft sensor is an alternative approach to obtain the membrane humidity online. Soft sensors have been widely used in the industrial process control to improve the quality of the product and assure safety in the production. The core of a soft sensor is to construct a soft sensing model. At a very general level, one can distinguish two types of soft sensors, namely, model driven soft sensor and data driven soft sensor.

Model driven soft sensors are based on equations describing mass-preservation principles, water balances, energy balances, and reaction kinetics underlying the PEM fuel cell process. There have been some studies of the model driven soft sensor of membrane humidity in PEM fuel cells. Pukrushpan et al. [11] developed a lumped parameter model for estimating relative humidity of the electrodes. A nonlinear estimator was developed for the estimation of membrane humidity and the pressure and inlet and outlet temperature at the electrodes. The estimation filter was also used to implement a feedback controller to regulate the excess oxygen ratio during changes in load [12]. Arcak et al. [13] developed a nonlinear observer for fuel-cell hydrogen estimation. Görgün et al. [14] developed an algorithm for estimating the membrane humidity in PEM fuel cells. Vepa [15] has applied nonlinear extended Kalman filtering to the estimates of the states of a PEM fuel cell.

However PEM fuel cell process is generally quite complex to model and are characterized by significant inherent nonlinearities, and model driven soft sensor may fail to provide satisfactory prediction performance when the process is operated under a wide range. Consequently, there has been an increasing interest toward the development of data-driven soft sensors using multivariate regression techniques. Because data-driven soft sensors are easy to develop and to implement online, they are potentially more attractive than model driven soft sensor. In this paper, we adopt data-driven soft sensors based on partial least squares (PLS) method because this method is one of the widely used regression techniques and its successful application to the development of soft sensors has been reported for different processes [16–19].

## 2. Soft Sensor Based on DPLS

### 2.1. Partial Least Squares (PLS) Regression

We briefly review the standard PLS in order to establish soft sensor model. Let *X* be the *n* × *m* data matrix of *m* predictors measured on *n* samples and *Y* the *n* × *p* multivariate response data matrix with *p* variables from the same *n* samples. We assume that both of *X* and *Y* are centered across the columns. PLS regression is typically based on the basic latent component decomposition.

Assume that *X* and *Y* are linearly related by

(1) $$\begin{array}{c} Y=XC+V, \end{array}$$

where *C* is the coefficient matrix and *V* is the noise matrix with appropriate dimensions. PLS regression is typically based on the basic latent component decomposition:

(2) $$\begin{array}{c} X=TP^{T}+E,\\ Y=TQ^{T}+F, \end{array}$$

where *T* ∈ *R* ^*n*×*K*^ is a matrix for the latent components and matrix *P* ∈ *R* ^*m*×*K*^ and *Q* ∈ *R* ^*p*×*K*^ are known as the loading matrices. Matrices *E* and *F* are the corresponding residual matrices with appropriate dimensions. Equation (2) formulates the PLS outer model. The final goal of PLS is to describe as much variation of *Y* as possible and at the same time get a useful relation between *X* and *Y*. The latent component matrix *T* is defined as

(3) $$\begin{array}{c} T=XW, \end{array}$$

where *W* ∈ *R* ^*m*×*K*^ is a *K* direction vectors, so that we have the prediction model

(4) $$\begin{array}{c} Y=X\beta +F. \end{array}$$

PLS requires finding the columns of *W* = (*w*_1_, *w*_2_,…, *w*_*K*_) from successive optimization problems. The criterion to find the *k*th direction vector *w*_*K*_ for univariate *Y* is formulated as

(5) wk=argmax⁡w {wTZTZw}subject  to wTw=1, wTSXw=0,

where *Z* = *Y* ^*T*^ *X* and *S*_*X*_ = *X* ^*T*^ *X*/*n* is the sample covariance matrix of *X*.

There are two main formulations for finding PLS direction vectors which were originally derived from an algorithm, known as nonlinear iterative partial least-squares NIPALS [15], without a specific optimization problem formulation. Subsequently, a statistically inspired modification of PLS, known as SIMPLS, was proposed with an algorithm by directly extending the univariate PLS formulation.

### 2.2. Dynamic PLS

The PLS regression implicitly assumes that they are independent between the current and past observations. This assumption is not valid in PEM fuel cell processes because the membrane humidity at an instant can result from the cumulative effects of past process conditions, such as temperature, pressure, and flow rate. Dynamic PLS is an extension of PLS which can be implemented by augmenting each observation vector with previous observations and stacking the predictor matrix in the following way:

(6) $$\begin{array}{c} X\left(h\right)=\left[\begin{array}{cccc} x_{t}^{T} & x_{t-1}^{T} & \cdots & x_{t-h}^{T}\\ x_{t-1}^{T} & x_{t-2}^{T} & \cdots & x_{t-h-1}^{T}\\ \vdots & \vdots & \ddots & \vdots \\ x_{t+h-n}^{T} & x_{t+h-n-1}^{T} & \cdots & x_{t-n}^{T} \end{array}\right], \end{array}$$

where *x*_*t*_^*T*^ is the *m*-dimensional observation vector in the training set at time instant *t* and *x*_*t*−1_^*T*^ is the *m*-dimensional observation vector in the training set at time instant *t* − 1. Using the predictor *X*(*h*) in PLS regression a dynamic PLS (DPLS) model will be obtained [14]. Including such time lags in the predictor data can provide information on the dynamic process conditions to the model.

### 2.3. Data Preprocessing

DPLS-based soft sensor is data dependent. As a consequence, it is very important for analyzing and preprocessing the data used for PLS model calibration and validation. First, abnormal data need to be detected and removed from the dataset, as they will result in a DPLS-based model which is not representative of the ordinary process behavior [17]. Second, a suitable scaling procedure needs to be adopted [15].

Measurement noise causes errors in model estimation and has therefore to be dealt with by increasing the signal to noise ratio (SNR) of the data. A data variable *x*_*k*_ is smoothed by using a weighted sum of previous measurements in a window of finite length (finite impulse response (FIR)):

(7) $$\begin{array}{c} x_{t,k}^{d}=\frac{1}{N}\cdot \sum_{i=1}^{N}a_{i}x_{t-i,k}\;\text{with}\;\;\sum_{i=1}^{N}a_{i}=1, \end{array}$$

where *N* is the filter length, *a*_*i*_ are the filter coefficients, and *x*_*d*_^*t*^ is the denoised sample. If all previous measurements are used then an infinite length (infinite impulse response (IIR)) filter is obtained. An example of an IIR filter is the exponentially weighted moving average (EWMA) filter that is recursively implemented as

(8) $$\begin{array}{c} x_{t,k}^{d}=\alpha x_{t,k}+\left(\alpha -1\right)x_{t-1,k}^{d}, \end{array}$$

where *α* is an adjustable smoothing parameter with values between 0 and 1.

Outliers can be defined as samples that are not consistent with the majority of the data. Generally, there are two ways to treat outliers. The first one relies on detection and replacement of outliers with some reasonable values while the second one uses advantages of robust techniques for model parameters estimation which are less affected by presence of outliers in the data set.

## 3. Hardware-in-the-Loop Test System

How to get the data of the membrane humidity is the most important problem when the DPLS-based soft sensing is applied to the estimation of the membrane humidity in PEM fuel cell. High-resolution soft X-ray radiography can be used to observe the liquid water in single cell [18]. A vanadium thin-film was used as the target material for generating soft X-rays. The cell was carefully fixed on a computer controlled four-axis stage that was able to move in the *X*, *Y*, and *Z* directions with a resolution of 1 mm and rotate around the *Z*-axis. The liquid water thickness was calculated using a dry image and an image with liquid water as references. Moreover, the liquid water thickness can be transformed into the membrane humidity and this method can be applied to the PEM fuel cell stack.

**Figure 1:**
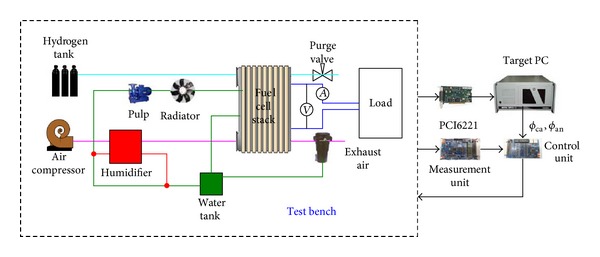
The Schematic diagram of HIL test system.

**Figure 2:**
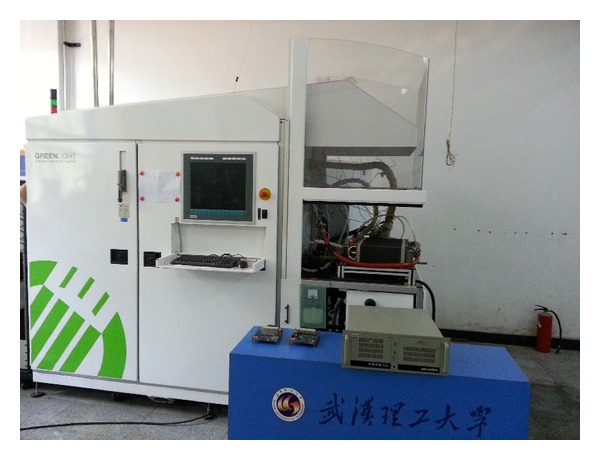
Real hardware-in-the-loop test system.

**Figure 3:**
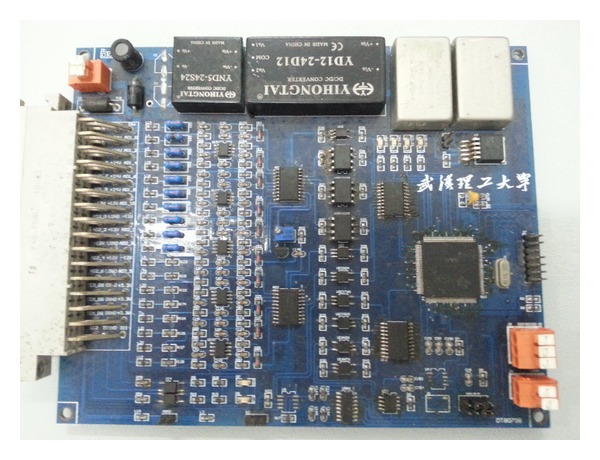
Measurement unit.

**Figure 4:**
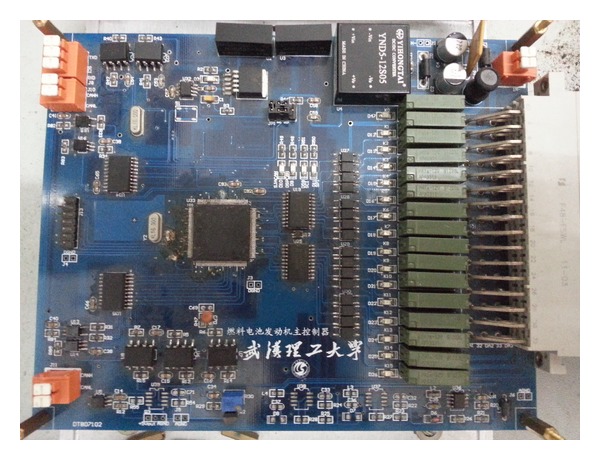
Control unit.

At present high-resolution soft X-ray radiography is not equipped in our laboratory. In order to obtain data of membrane humidity a hardware-in-the-loop (HIL) test system is set up. HIL test system is a well-established technique and increasingly being used as cost-effective means for rapid prototyping, integration, and validation of complex engineering systems. HIL test system is a real-time test methodology where real subsystem parts of a complex engineering system are coupled together with the numerical models of the remaining subsystems to form its complete representation. The schematic diagram of HIL test system is shown in Figure 1. In Figure 1 the test bench is FCATS G500 (GreenLight In. Co., Canada). The measurement unit is used to gather signals from FCATS G500. The control unit has two functions. One is to estimate the relative humidity *ϕ*_an_ and *ϕ*_ca_, the others are to control the flow rate of cooling water *W*_cool_, flow rate of compressed air *W*_cp_, and flow rate of humidifier *W*_inj_. The measurement unit and the control unit are developed by out laboratory. Based on the xPC Target product of MATLAB Target PC is used to obtain data of membrane humidity. There is a board, which is PCI6221 produced by National Instruments, in the industrial control computer to acquire data from FCATS G500. The water balance model of PEM fuel cell is built in the Target PC by using MATLAB, Simulink, and Real-Time Workshop. Target PC and the control unit are communicating through controller area network (CAN) protocol. The real HIL test system is shown in Figure 2 and the real measurement unit and control unit are shown in Figures 3 and 4.

The relative humidity in anode and cathode *ϕ*_an_ and *ϕ*_ca_ is calculated by

(9) $$\begin{array}{c} \phi_{\text{an}}=\frac{P_{v,\text{an}}}{P_{\text{sat,an}}},\;\;\phi_{\text{ca}}=\frac{P_{v,\text{ca}}}{P_{\text{sat,ca}}}, \end{array}$$

where *P*_*v*,an_ and *P*_*v*,ca_ are the partial pressure of vapor (kPa) in anode and cathode and *P*_sat,an_ and *P*_sat,ca_ are the partial pressure (kPa) of saturation vapor in anode and cathode.

According to ideal gas law, the partial pressure of vapor in anode and cathode *P*_*v*,an_ and *P*_*v*,ca_ are calculated by

(10) $$\begin{array}{c} p_{v,\text{an}}=\frac{\left(m_{v,\text{an}}R_{v}T_{\text{st}}\right)}{V_{\text{an}}},\\ p_{v,\text{ca}}=\frac{\left(m_{v,\text{ca}}R_{v}T_{\text{st}}\right)}{V_{\text{ca}}}, \end{array}$$

where *m*_*v*,an_ and *m*_*v*,ca_ are the vapor mass (kg) in the anode and cathode, respectively, *R*_*v*_ is vapor gas constant, *T*_st_ is temperature (K) in fuel cell stack, and *V*_an_ and *V*_ca_ are the volume (cm^3^) in the anode and cathode.

The water masses in the anode and cathode are given by

(11) $$\begin{array}{c} \frac{dm_{v,\text{an}}}{dt}=W_{v,\text{an},\text{in}}-W_{v,\text{an},\text{out}}-W_{v,\text{mem}},\\ \frac{dm_{v,\text{ca}}}{dt}=W_{v,\text{ca},\text{in}}-W_{v,\text{ca},\text{out}}+W_{{\text{H}}_{2}\text{O},\text{gen}}+W_{v,\text{mbr}}+W_{\text{inj}}, \end{array}$$

where *W*_*v*,mbr_ is the water transport through the membrane from the anode to the cathode, *W*_inj_ is the mass flow rate of injected water from humidifier, *W*_*v*,an,in_ and *W*_*v*,ca,in_ are the mass flow rate of inlet vapor in the anode and cathode, *W*_*v*,an,out_ and *W*_*v*,ca,out_ are the mass flow rate of outlet vapor in the anode and cathode, and *W*_H_2_O,gen_ is the mass flow rate of water generated in the fuel cell stack.

The governing equation for hydrogen, oxygen, and nitrogen can be written as [8]

(12) $$\begin{array}{c} \frac{dm_{{\text{H}}_{2}}}{dt}=W_{{\text{H}}_{2},\text{in}}-W_{{\text{H}}_{2},\text{out}}-W_{{\text{H}}_{2},\text{rct}},\\ \frac{dm_{{\text{O}}_{2}}}{dt}=W_{{\text{O}}_{2},\text{in}}-W_{{\text{O}}_{2},\text{out}}-W_{{\text{O}}_{2},\text{rct}},\\ \frac{dm_{{\text{N}}_{2}}}{dt}=W_{{\text{N}}_{2},\text{in}}-W_{{\text{N}}_{2},\text{out}}, \end{array}$$

where *W*_H_2_,in_ is flow rate of inlet hydrogen, *W*_H_2_,out_ is flow rate of outlet hydrogen, *W*_N_2_,in_ is flow rate of inlet nitrogen, *W*_N_2_,out_ is flow rate of outlet nitrogen, *W*_H_2_,rct_ and *W*_O_2_,rct_ are the mass flow rate of hydrogen and oxygen consumed in the reaction respectively, *W*_O_2_,in_ is flow rate of inlet oxygen, and *W*_O_2_,out_ is flow rate of outlet oxygen.

In the cathode, inlet mass of oxygen, nitrogen, and vapor is related to mass flow rate and humidity of inlet air:

(13) $$\begin{array}{c} W_{{\text{O}}_{2},\text{in}}=\frac{y_{{\text{O}}_{2}}}{\left(1+\Omega_{\text{atm}}\right)W_{\text{cp}}},\\ W_{{\text{N}}_{2,\text{in}}}=\frac{y_{{\text{N}}_{2}}}{\left(1+\Omega_{\text{atm}}\right)W_{\text{cp}}},\\ W_{v,\text{ca},\text{in}}=\frac{\Omega_{\text{atm}}}{\left(1+\Omega_{\text{atm}}\right)W_{\text{cp}}}, \end{array}$$

where *W*_cp_ is flow rate of inlet air by using compressor, *y*_O_2__ and *y*_N_2__ are oxygen mole fraction and nitrogen mole fraction of dry air, and *Ω*_atm_ is humidity ratio of air.

The pressure of cathode is the sum of the partial pressure of oxygen, nitrogen, and vapor:

(14) $$\begin{array}{c} p_{\text{ca}}=p_{{\text{O}}_{2}}+p_{{\text{N}}_{2}}+p_{v,\text{ca}}. \end{array}$$

According to ideal gas law, the partial pressure of oxygen, nitrogen, and vapor is

(15) $$\begin{array}{c} p_{{\text{O}}_{2}}=\frac{\left(m_{{\text{O}}_{2}}R_{{\text{O}}_{2}}T_{\text{st}}\right)}{V_{\text{ca}}},\\ p_{{\text{N}}_{2}}=\frac{\left(m_{{\text{N}}_{2}}R_{{\text{N}}_{2}}T_{\text{st}}\right)}{V_{\text{ca}}}, \end{array}$$

where *R*_O_2__ is oxygen gas constant and *R*_N_2__ is nitrogen gas constant.

Outlet gas flow rate in cathode is calculated by

(16) $$\begin{array}{c} W_{\text{ca},\text{out}}=k_{\text{ca},\text{out}}\left(P_{\text{ca}}-P_{\text{rm},\text{ca}}\right), \end{array}$$

where *P*_rm,ca_ is the pressure of return manifold in cathode.

The mass flow rate of each species out of the cathode is calculated as

(17) $$\begin{array}{c} W_{{\text{O}}_{2},\text{out}}=W_{\text{ca}}\cdot \frac{m_{{\text{O}}_{2}}}{m_{\text{ca}}},\\ W_{{\text{N}}_{2},\text{out}}=W_{\text{ca}}\cdot \frac{m_{{\text{N}}_{2}}}{m_{\text{ca}}},\\ W_{v,\text{ca},\text{out}}=\frac{\left(p_{v,\text{ca}}V_{\text{ca}}M_{v}\right)}{\left(RT_{\text{st}}m_{\text{ca}}\right)}\cdot W_{\text{ca}}, \end{array}$$

where *m*_ca_ = *m*_O_2__ + *m*_N_2__ + (*p*_*v*,ca_ *V*_ca_ *M*_*v*_)/(*RT*_st_) is the total mass of the cathode gas.

The mass flow rate of hydrogen and oxygen consumed in the reaction and water generated in the reaction is calculated by

(18) $$\begin{array}{c} W_{{\text{O}}_{2},\text{rct}}=\frac{M_{{\text{O}}_{2}}\left(nI_{\text{st}}\right)}{\left(4F\right)},\\ W_{{\text{H}}_{2},\text{rct}}=\frac{M_{{\text{H}}_{2}}\left(nI_{\text{st}}\right)}{\left(2F\right)},\\ W_{v,\text{gen}}=\frac{M_{{\text{H}}_{2}\text{O}}\left(nI_{\text{st}}\right)}{\left(2F\right)}, \end{array}$$

where *I*_st_ is the stack current.

The outlet hydrogen and vapor mass flow rate is calculated by the following equations:

(19) $$\begin{array}{c} W_{{\text{H}}_{2},\text{an},\text{out}}=\frac{1}{\left(1+\Omega_{\text{an},\text{out}}\right)W_{\text{an},\text{out}}},\\ W_{v,\text{an},\text{out}}=\frac{\varphi_{\text{an},\text{out}}}{\left(1+\Omega_{\text{an},\text{out}}\right)W_{\text{an},\text{out}}},\\ \Omega_{\text{an},\text{out}}=\frac{M_{v}}{M_{{\text{H}}_{2}}}\cdot \frac{\left(m_{{\text{H}}_{2}}R_{{\text{H}}_{2}}\right)}{\left(m_{v,\text{an}}R_{{\text{H}}_{2}}\right)}. \end{array}$$

The water transport across the membrane is achieved through two distinct phenomena: electro-osmotic and back-diffusion. Combining the two water transport mechanisms, the water transport through the membrane is given by

(20) $$\begin{array}{c} W_{v,\text{mem}}=M_{v}A_{\text{fc}}n\left(\frac{n_{d}I_{\text{st}}}{F}-D_{w}\cdot \frac{\left(c_{v,\text{an}}-c_{v,\text{an}}\right)}{t_{m}}\right). \end{array}$$

The electro-osmotic drag coefficient *n*_*d*_ and the diffusion coefficient *D*_*w*_ vary with humidity in the membrane [8, 20]:

(21) $$\begin{array}{c} n_{d}=0.0029\lambda_{m}^{2}+0.05\lambda_{m}-3.4\times 10^{-19},\\ D_{w}=D_{\lambda }\exp\left(2416\left(\frac{1}{303}-\frac{1}{T_{\text{st}}}\right)\right), \end{array}$$

where

(22) $$\begin{array}{c} D_{\lambda }=\{\begin{array}{cc} 10^{-6}, & \lambda_{m}<2\\ 10^{-6}\left(1+2\left(\lambda_{m}-2\right)\right), & 2\leq \lambda_{m}\leq 3\\ 10^{-6}\left(3-1.67\left(\lambda_{m}-3\right)\right), & 3<\lambda_{m}<4.5\\ 1.25\times 10^{-6}, & \lambda_{m}\geq 4.5. \end{array} \end{array}$$

Membrane average water content *λ*_*m*_ is calculated by [8]

(23) $$\begin{array}{c} \lambda_{m}=\{\begin{array}{cc} 0.043+17.81\phi_{m}-39.85\phi_{m}^{2}+36.0\phi_{m}^{3}, & 0<\phi_{m}\leq 1\\ 14+1.4\left(\phi_{m}-1\right), & 1<\phi_{m}\leq 3, \end{array} \end{array}$$

where

(24) $$\begin{array}{c} \phi_{m}=\frac{\left(\phi_{\text{an}}+\phi_{\text{ca}}\right)}{2}. \end{array}$$

## 4. Experimental Results 

**Figure 5:**
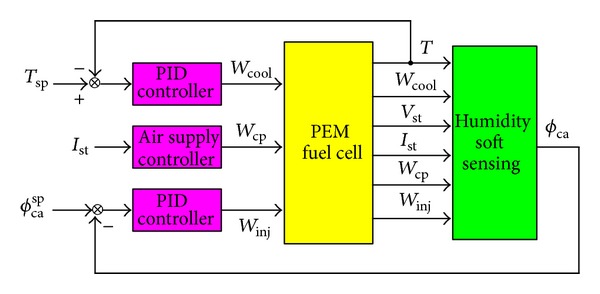
The schematic diagram of the relative humidity's soft sensor.

In this section the DPLS-based soft sensor is applied to the membrane humidity prediction in PEM fuel cell. The schematic diagram of the membrane humidity's soft sensor is shown in Figure 5. In Figure 5 stack temperature *T* and flow rate of humidifier *W*_inj_ are controlled by PID controller. The control method of flow rate of compressed air *W*_cp_ is the feedforward control. Soft sensing of the membrane humidity can be carried out by using stack temperature *T*, stack current *I*_st_, stack voltage *V*_st_, flow rate of cooling water *W*_cool_, flow rate of compressed air *W*_cp_, and water flow rate of humidifier *W*_inj_.

**Figure 6:**
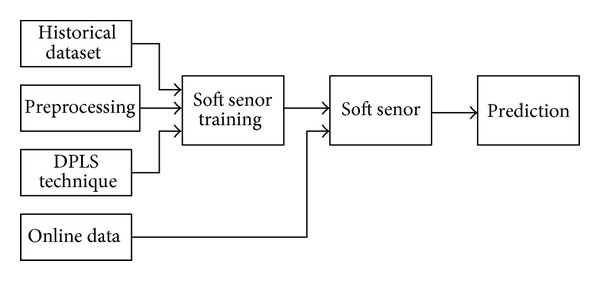
Flow diagram of the proposed soft sensor.

**Figure 7:**
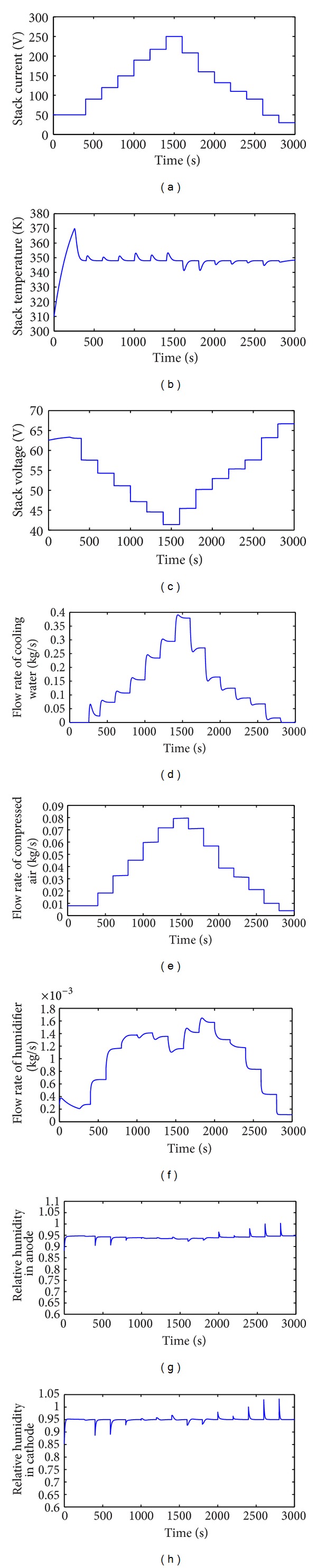
Some measurements of dataset which train DPLS-based soft sensor when *ϕ*_ca_ is set as 0.95. (a) Stack current *I*_st_; (b) stack Temperature *T*_stack_; (c) stack voltage *V*_st_; (d) flow rate of cooling water *W*_cool_; (e) flow rate of compressed air; (f) water flow rate of humidifier *W*_inj_; (g) relative humidity in anode; (h) relative humidity in cathode.

Flow diagram of the proposed soft sensor is shown in Figure 6. The development of practical online prediction soft sensors consists of two stages: training and online prediction. The historian dataset which trains DPLS-based soft sensor includes stack temperature *T*, stack current *I*_st_, stack voltage *V*_st_, flow rate of cooling water *W*_cool_, flow rate of compressed air *W*_cp_, water flow rate of humidifier *W*_inj_, and relative humidity in cathode and anode *ϕ*_ca_, *ϕ*_an_. Some measurements of the dataset which are used to train DPLS-based soft sensor are shown in Figure 7. In Figure 7(a) stack current *I*_st_ stepped up or down for every 200 seconds and the sample time is 0.2 seconds. Relative humidity in cathode *ϕ*_ca_ is set as 0.95 and stack's temperature is set as 65°C. The training dataset is obtained by measuring different *T*, *I*_st_, *W*_cool_, *W*_cp_, and *ϕ*_ca_.

To investigate the fitting and predicting ability of a DPLS model, the root-mean-square error of calibration indicates the fit of the model to the calibration data [16]. It is defined as

**Figure 8:**
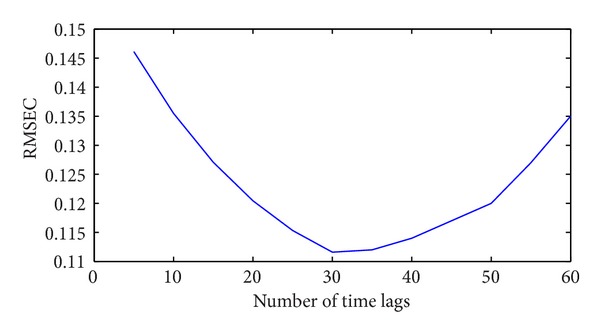
The RMSEC for different numbers of time lags of the DPLS algorithm.

(25) RMSEC=∑i=1n(y^i−yi)2n,

where the y^i is the value of the predicted variable when all samples are included in the model formation and *n* is the number of calibration samples. The RMSEC for different numbers of time lags of the DPLS algorithm is shown in Figure 8. The time lag of the DPLS-based soft sensor is selected as 30. In the training stage DPLS is implemented by using CVX software [21].

**Figure 9:**
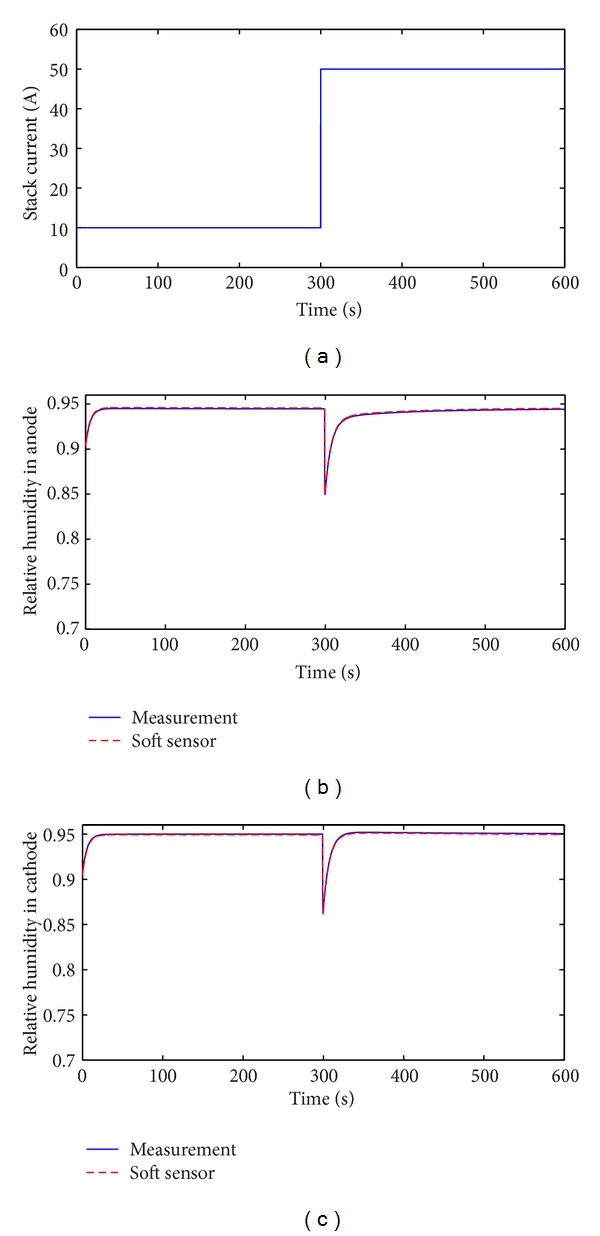
Comparison of measured and soft sensor's relative humidity with stepped change of stack current. (a) Stack current *I*_st_; (b) anode; (c) cathode.

After the parameters of DPLS-based soft sensor are trained this soft sensor is applied to predict *ϕ*_ca_ and *ϕ*_an_. The prediction of *ϕ*_ca_ and *ϕ*_an_ under the condition of stepped current is shown in Figure 9. The stack current is stepped from 10 A to 50 A in the instance of 300 seconds. The figure indicates that the proposed DPLS based soft sensor performs well and can estimate *ϕ*_ca_ and *ϕ*_an_ accurately.

**Figure 10:**
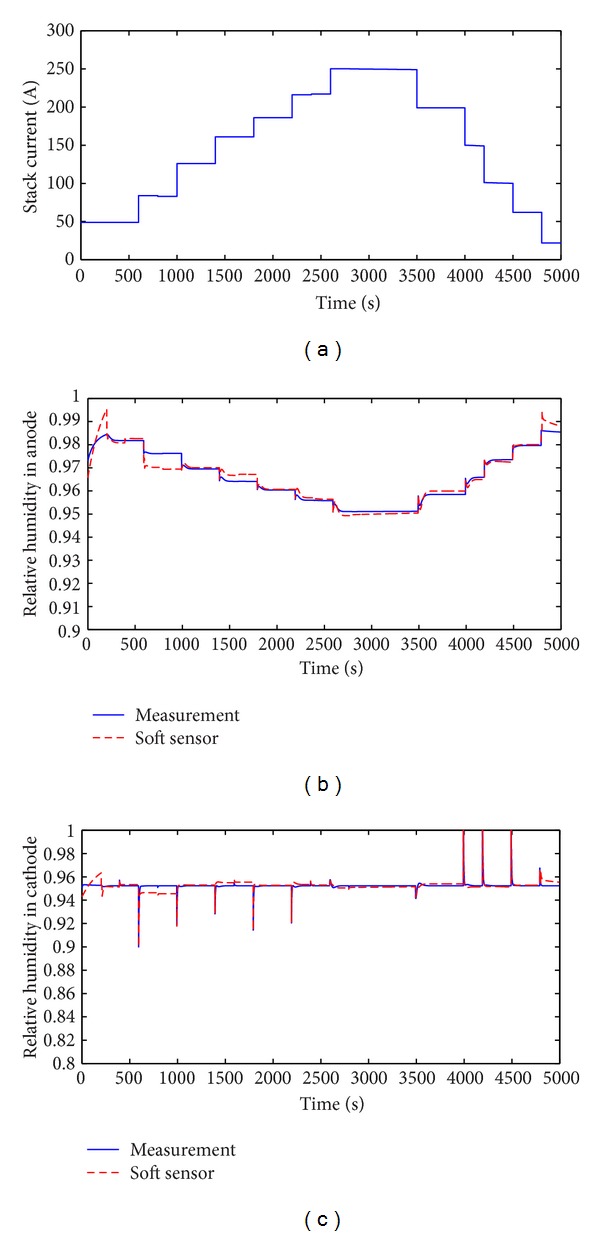
Comparison of measured and soft sensor's relative humidity with multiple stepped change of stack current. (a) Stack current *I*_st_; (b) anode; (c) cathode.

Moreover, the DPLS-based soft sensor is tested by the continuous change of the stack currents. During 0–600-second time period stack current is 50 A, steps up to 80 A during 600–1000-second time period, steps up to 130 A during 1000–1400-second time period, steps up to 160 A during 1400–1800-second time period, steps up to 180 A during 1800–2200-second time period, steps up to 220 A during 2200–2600-second time period, steps up to 250 A during 2600–3500-second time period, steps down to 200 A during 3500–4000-second time period, steps down to 150 A during 4000–4200-second time period, steps down to 100 A during 4200–4500-second time period, steps down to 60 A during 4500–4800-second time period, and steps down to 20 A during 4800–5000-second time period. Experimental result is shown in Figure 10. The error between measured relative humidity and soft sensor's relative humidity with multiple stepped change of stack current is less than 0.15. These results demonstrate that DPLS-based soft sensor is available to predict humidity of anode and cathode in the continuous change of the stack currents.

**Figure 11:**
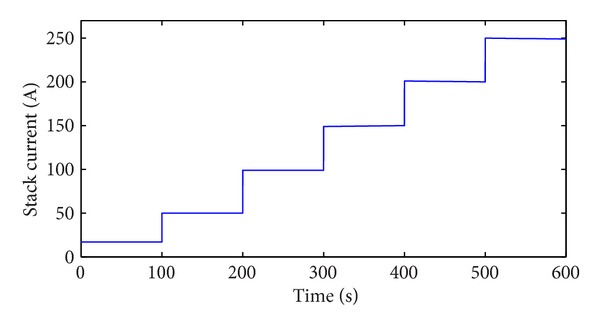
Stack current in the speed-up condition.

**Figure 12:**
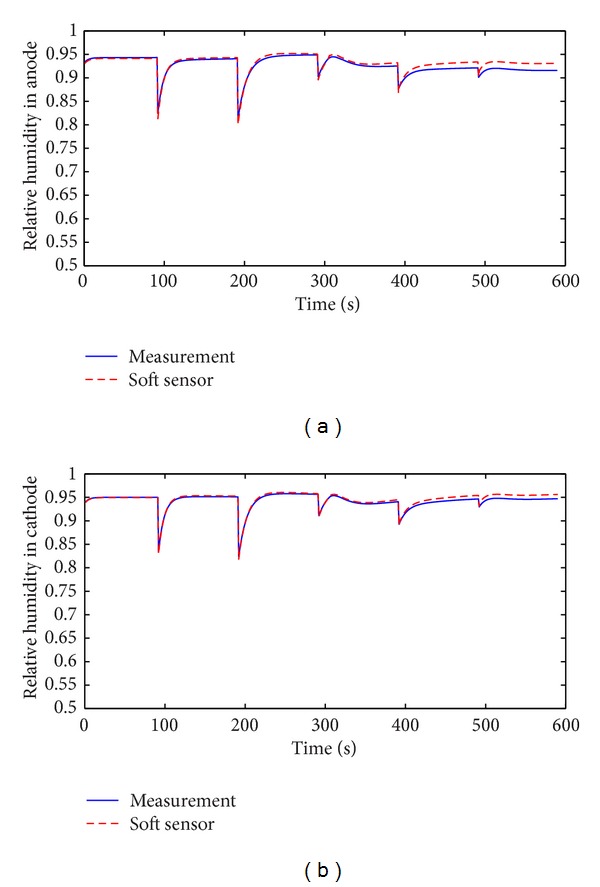
Comparison of measured and soft sensor's relative humidity in the speed-up condition when relative humidity of cathode is set as 0.95. (a) Anode; (b) cathode.

**Figure 13:**
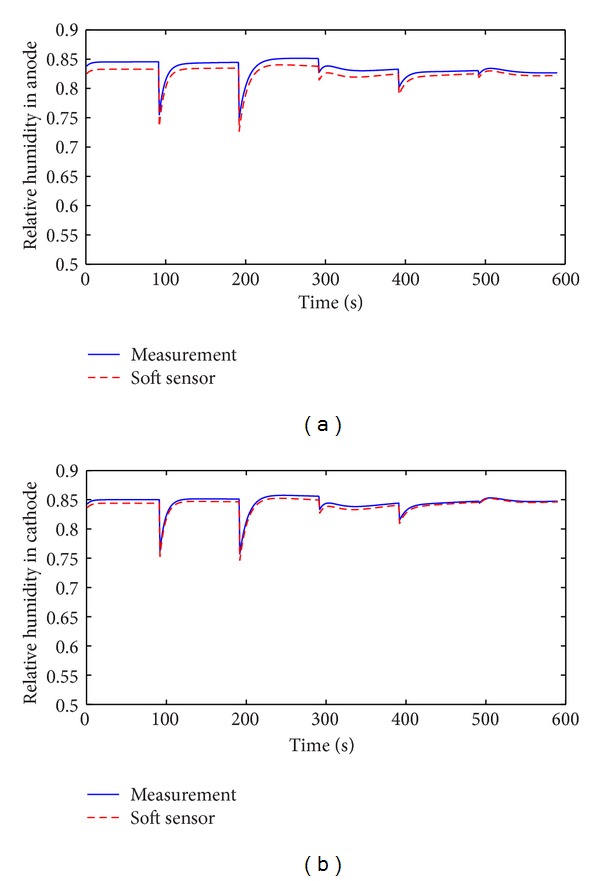
Comparison of measured and soft sensor's relative humidity in the speed-up condition when relative humidity of cathode is set as 0.85. (a) Anode; (b) cathode.

**Figure 14:**
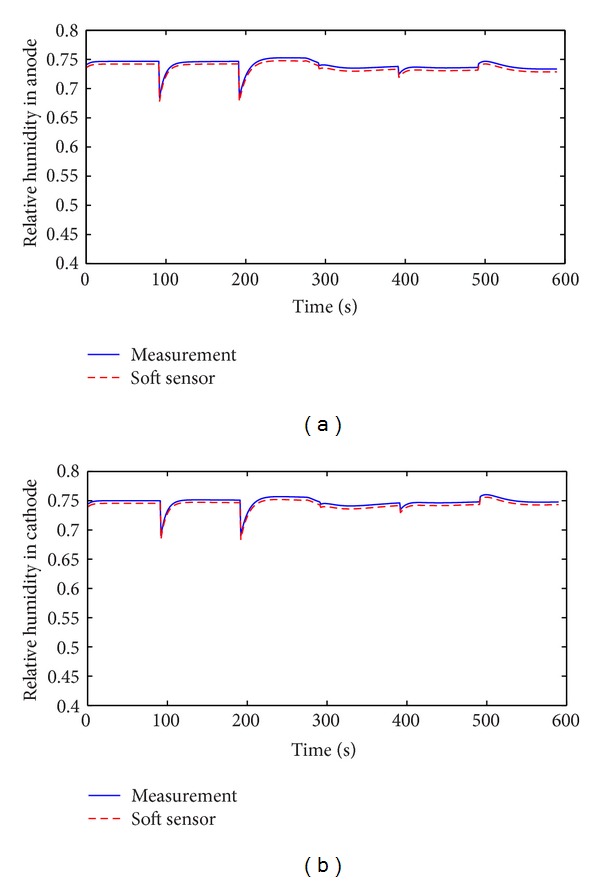
Comparison of measured and soft sensor's relative humidity with rapid start when relative humidity of cathode is set as 0.75. (a) Anode; (b) cathode.

**Figure 15:**
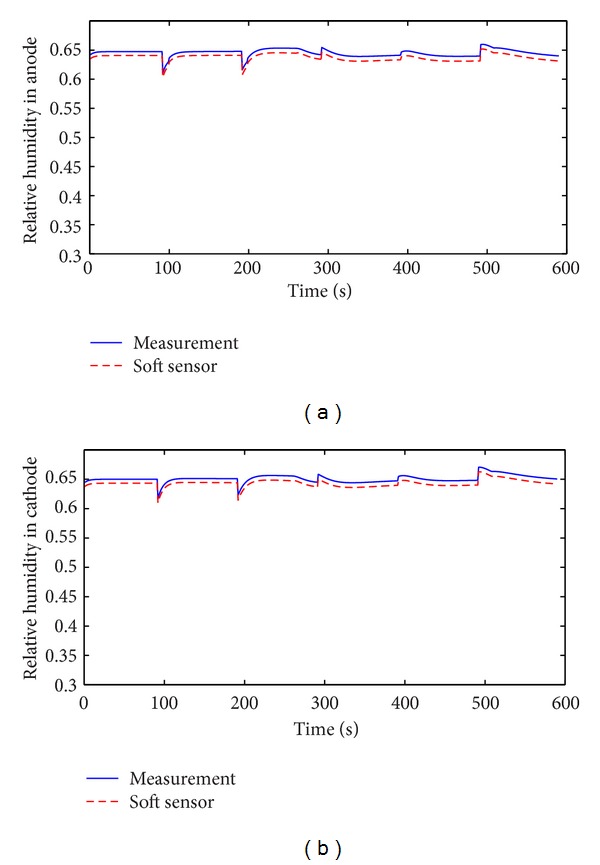
Comparison of measured and soft sensor's relative humidity with rapid start when relative humidity of cathode is set as 0.65. (a) Anode; (b) cathode.

Speed-up is an important working condition when PEM fuel cell is applied to automobile. The soft sensor is tested in the speed-up condition and the typical change of stack current is shown in Figure 11. Figures 12, 13, 14, and 15 show the comparison of measured and soft sensor's relative humidity in the speed-up conditions when relative humidity in cathode is set from 0.95 to 0.65. These results demonstrate that DPLS-based soft sensor is available to predict membrane humidity in the speed-up condition when relative humidity of cathode is set in different humidity. All these experimental results show that the proposed DPLS-based soft sensor is feasible to estimate the humidity in PEM fuel cell and is an effective way to control relative humidity in cathode.

## 5. Conclusions 

In this study, a DPLS-based soft sensor was developed for a PEM fuel cell in order to predict the membrane humidity. In order to obtain dataset which trains the soft sensor a HIL test system is constructed and demonstrates the feasibility and accuracy of DPLS-based soft sensor. The DPLS-based soft sensor is tested in different working conditions of PEM fuel cell. Experimental results display that the proposed DPLS-based soft sensor is feasible to estimate the humidity in PEM fuel cell and is an effective way to control relative humidity in cathode.
